# Supplementary material for: Liposomal nanotheranostics for multimode targeted in vivo bioimaging and near‐infrared light mediated cancer therapy
Source: Commun Biol. 2020 Jun 5;3:284. doi: 10.1038/s42003-020-1016-z (PMC7275035; doi:10.1038/s42003-020-1016-z)
Supplement: Supplementary file 1 — Supplementary Information [file 42003_2020_1016_MOESM1_ESM.pdf]

Supporting information

Liposomal Nanotheranostics for Multimode Targeted *In Vivo* Bioimaging and Near-Infrared Light Mediated Cancer Therapy

Table of Contents

Supporting Figures

Supplementary Figure 1. Low beam voltage (100 kV) transmission electron microscopic (TEM) images, particle size distribution measured through dynamic light scattering (DLS) measurement and time dependent aqueous dispersion of various formulations of designed liposome based nanotheranostics named as NFGL nanohybrids.

Supplementary Figure 2. (a, b) TEM images of synthesized fluorescent graphene quantum dots (GQDs) at different scale bar, calculated size distribution shown in inset, (c) AFM image of graphene quantum dots with height profile and (d) TEM image of polymer stabilized gold nanoparticles.

Supplementary Figure 3. Energy Dispersive X-Ray Analysis (EDAX) of designed NFGL nanohybrid.

Supplementary Figure 4. Zeta potential measurement of graphene quantum dots (GQDs), polymer stabilized gold nanoparticles (AuNPs), liposomes and NFGL.

Supplementary Figure 5. RAMAN spectra of graphene quantum dots (GQDs) and NFGL nanohybrid.

Supplementary Figure 6. Absorbance of NFGL nanohybrid loaded with anticancer drug doxorubicin hydrochloride in various conditions.

Supplementary Figure 7. Time dependent photothermal transduction measurements of GQDs loaded nanohybrids and NFGL nanohybrids at various concentrations.

Supplementary Figure 8. % Drug release pattern of designed DOX-NFGL nanohybrid.

Supplementary Figure 9. The effect of NIR light for disintegration/or degradation of NFGL nanohybrid.

Supplementary Figure 10. (a, b) FTIR spectra of GQDs-Liposome-FA, NFGL-FA, DOX-NFGL-FA, GQDs-Liposome, NFGL and DOX-NFGL nanohybrids.

Supplementary Figure 11. Cancer cell imaging and cellular uptake of NFGL nanohybrid with and without folic acid attachment.

Supplementary Figure 12. (a) Production of reactive oxygen species (ROS) during NIR light exposure, (b, c) electron resonance spectra (ESR) of NFGL nanohybrid before and after NIR light treatment.

Supplementary Figure 13. (a) Qualitative and (b) quantitative analysis (* $P \leq 0.05$) of ROS from 4T1 cancer cells treated NFGL nanohybrid in various conditions. C+NIR is NIR treated cells, C+NFGL is NFGL nanohybrids treated cells, C+NFGL+NIR is NFGL nanohybrids treated cells under NIR light exposure.

Supplementary Figure 14. NIR light mediated in vitro therapeutics efficiencies (% cell viability measured through MTT assay, *p, **p < 0.05, < 0.01) of NFGL nanohybrid and its various components in different conditions.

Supplementary Figure 15. Therapeutics measurement of designed NFGL nanohybrids and various components of NFGL nanohybrid on MCF-7 cancer cells in various conditions. (% cell viability measured through MTT assay, *** $P \leq 0.001$ and **** $P \leq 0.0001$).

Supplementary Figure 16. Localized tumor diagnosis of 4T1 breast tumor in mice body using NFGL-FA nanohybrid followed by IVIS imaging and X-ray CT imaging modalities before and after NIR light exposure (750 nm, 1 W for 10 minutes) at various time points (1 h, 6 h and 48 h) of post injection. In both imaging modalities, pre-injected mice are considered as control groups.

Supplementary Figure 17. (a) Time dependent emission intensity measurement from 4T1 tumor after intra-venous injection of NFGL-FA and (b) specific bio-distribution analysis of NFGL-FA after intravenous injection in 4T1 tumor bearing mice.

Supplementary Figure 18. (a) Hematoxylin and Eosin (H&E) measurements of major organs at various time of post-injection of NFGL-FA nanohybrid and (b) body weight measurements of different mice groups during various therapeutic conditions (different formulations of NFGL nanohybrid is injected intravenously in 4T1 tumor bearing mice).

Supporting Tables

Supplementary Table 1. Quantitative measurements of radiodensity (HU values) in major organs and tumor form X-ray CT imaging with and without NIR exposure experiments and compared with pre-injected mice.

Supplementary Table 2. Quantitative measurements of IVIS intensity in major organs and tumor with and without NIR exposure experiments and compared with pre-injected mice.

Supporting Equation

Supplementary equation 1. % Encapsulation efficiency of nanohybrids.

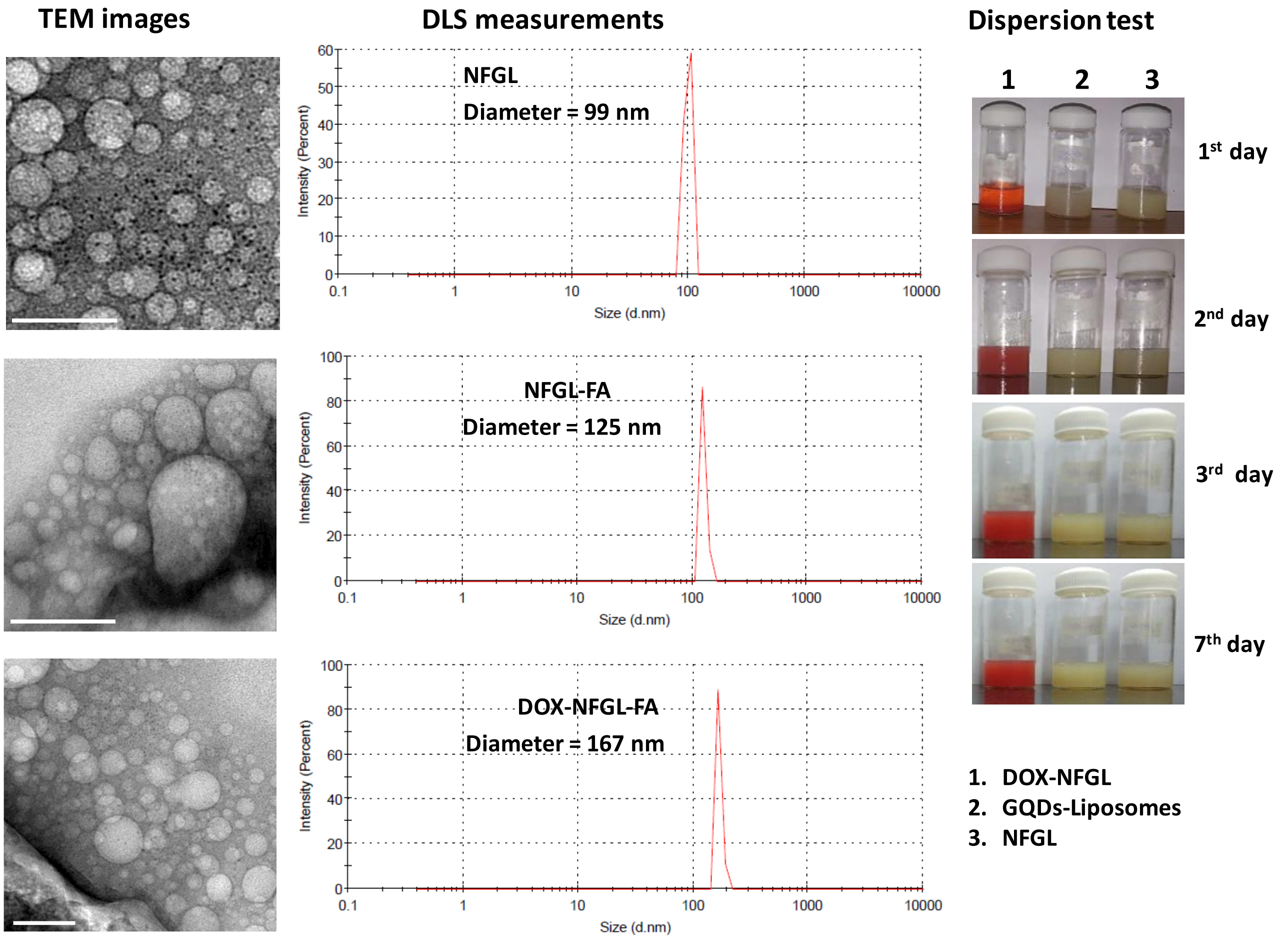

Supplementary Figure 1. Low beam voltage (100 kV) transmission electron microscopic (TEM) images (scale bar = 50 nm, 100 nm and 50 nm), particle size distribution measured through dynamic light scattering (DLS) measurement and time dependent aqueous dispersion of various formulations of designed liposome based nanotheranostics named as NFGL nanohybrid.

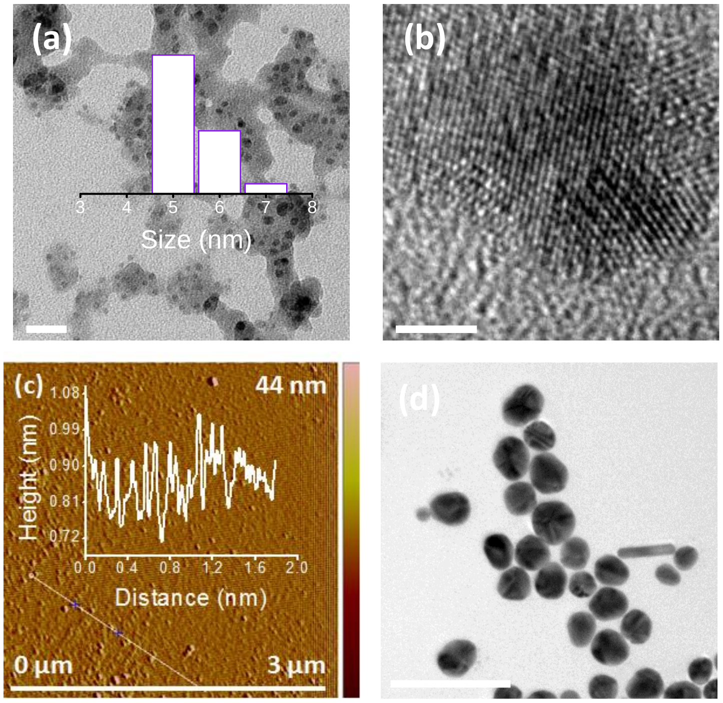

Supplementary Figure 2. (a, b) TEM images of synthesized fluorescent graphene quantum dots (GQDs) (scale bar = 20 nm and 2 nm), calculated size distribution shown in inset, (c) AFM image of graphene quantum dots with height profile and (d) TEM image of polymer stabilized gold nanoparticles (scale bar = 100 nm).

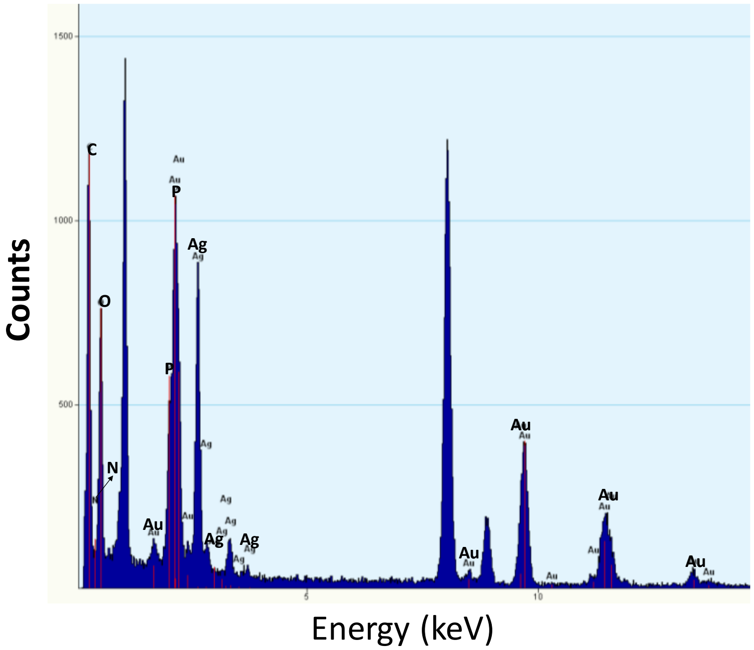

Supplementary Figure 3. Energy Dispersive X-Ray Analysis (EDAX) of designed NFGL nanohybrid.

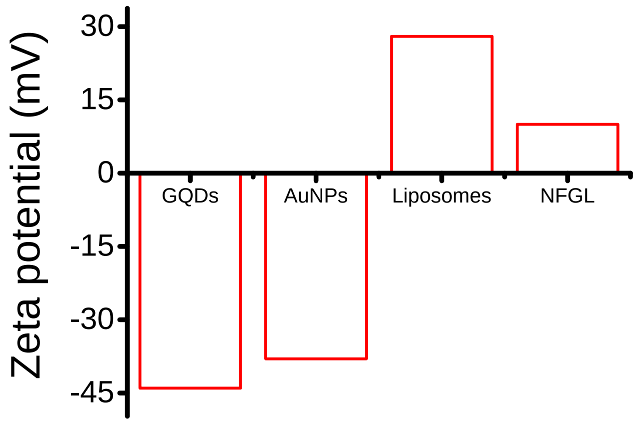

Supplementary Figure 4. Zeta potential measurement of graphene quantum dots (GQDs), polymer stabilized gold nanoparticles (AuNPs), liposomes and NFGL.

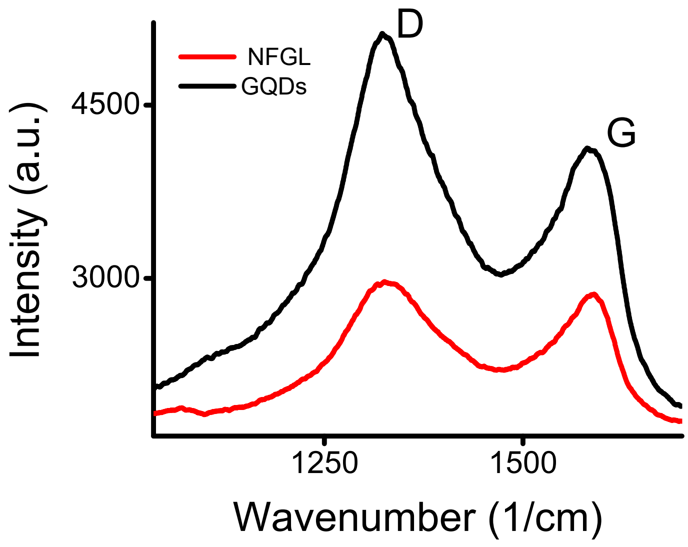

Supplementary Figure 5. RAMAN spectra of graphene quantum dots (GQDs) and NFGL nanohybrid.

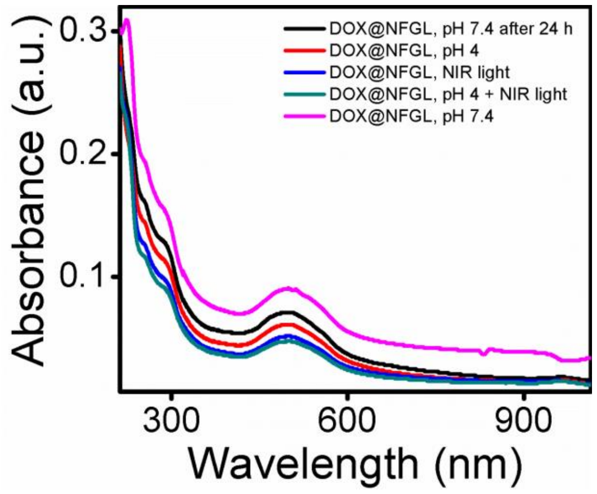

Supplementary Figure 6. Absorbance of NFGL nanohybrid loaded with anticancer drug doxorubicin hydrochloride in various conditions.

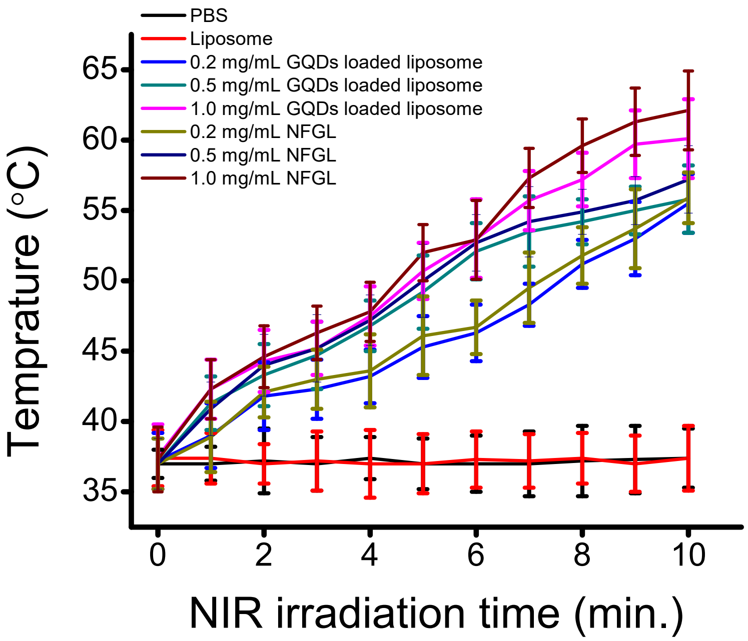

Supplementary Figure 7. Time dependent photothermal transduction measurements of GQDs loaded liposome and NFGL nanohybrids at various concentrations (n = 3).

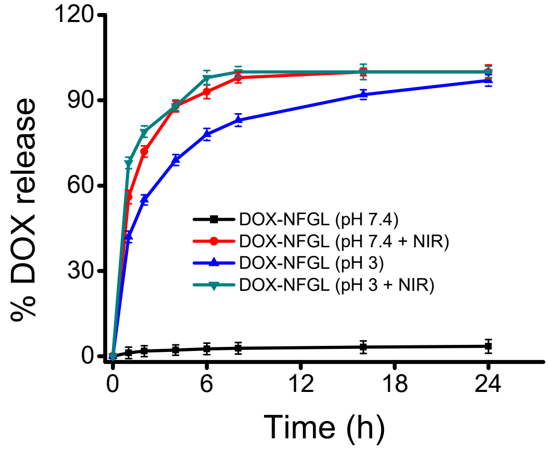

Supplementary Figure 8. % Drug release pattern of designed DOX-NFGL nanohybrids (n = 3).

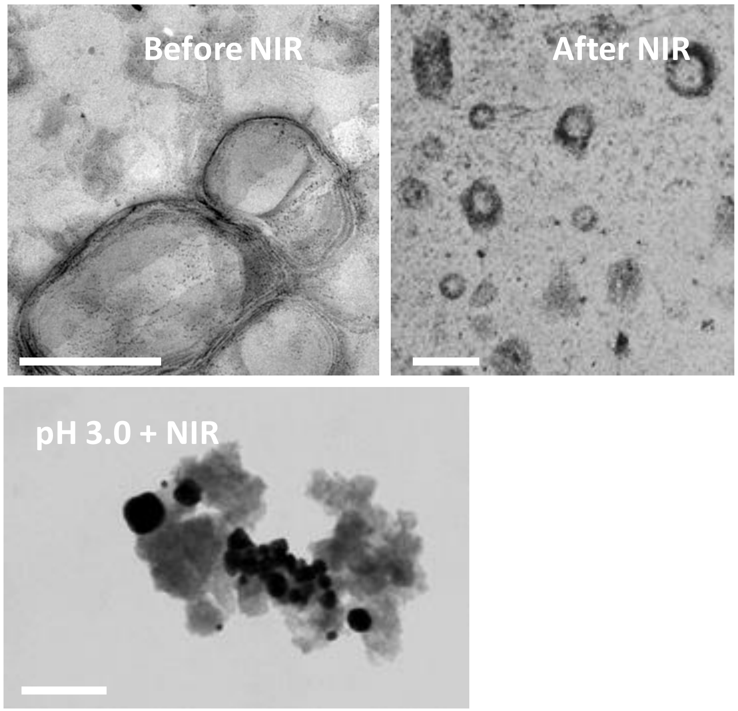

Supplementary Figure 9. The effect of NIR light for disintegration/or degradation of NFGL nanohybrids (scale bar = 500 nm, 200 nm and 200 nm).

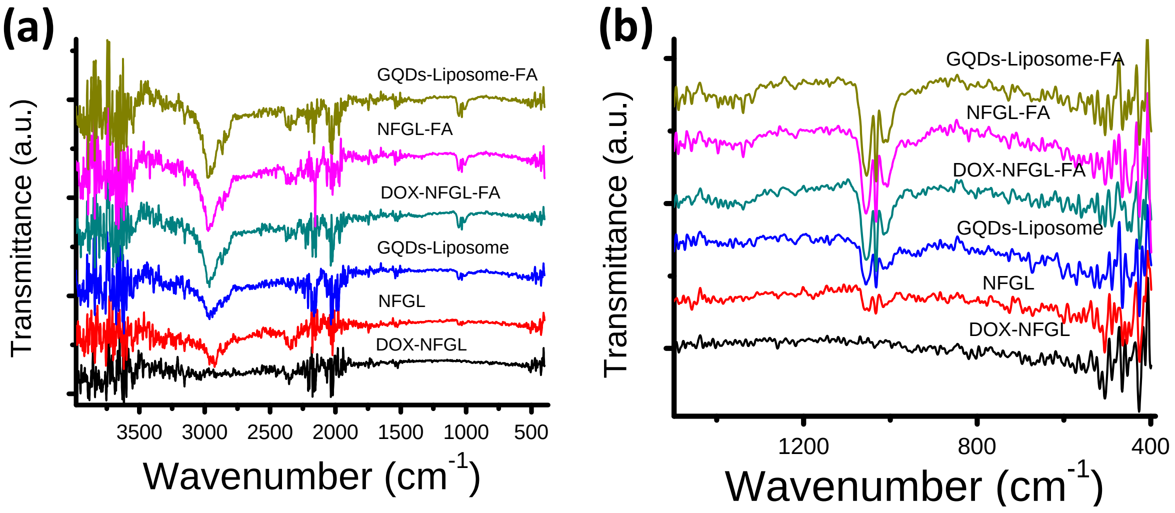

Supplementary Figure 10. (a, b) FTIR spectra of GQDs-Liposome-FA, NFGL-FA, DOX-NFGL-FA, GQDs-Liposome, NFGL and DOX-NFGL nanohybrids.

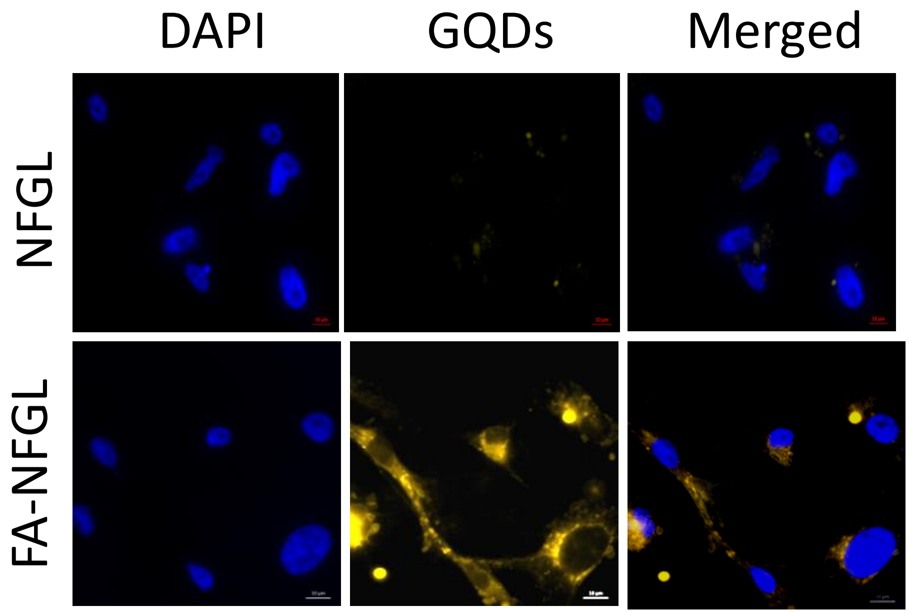

Supplementary Figure 11. Cancer cell imaging and cellular uptake of NFGL nano-hybrid with and without folic acid attachment.

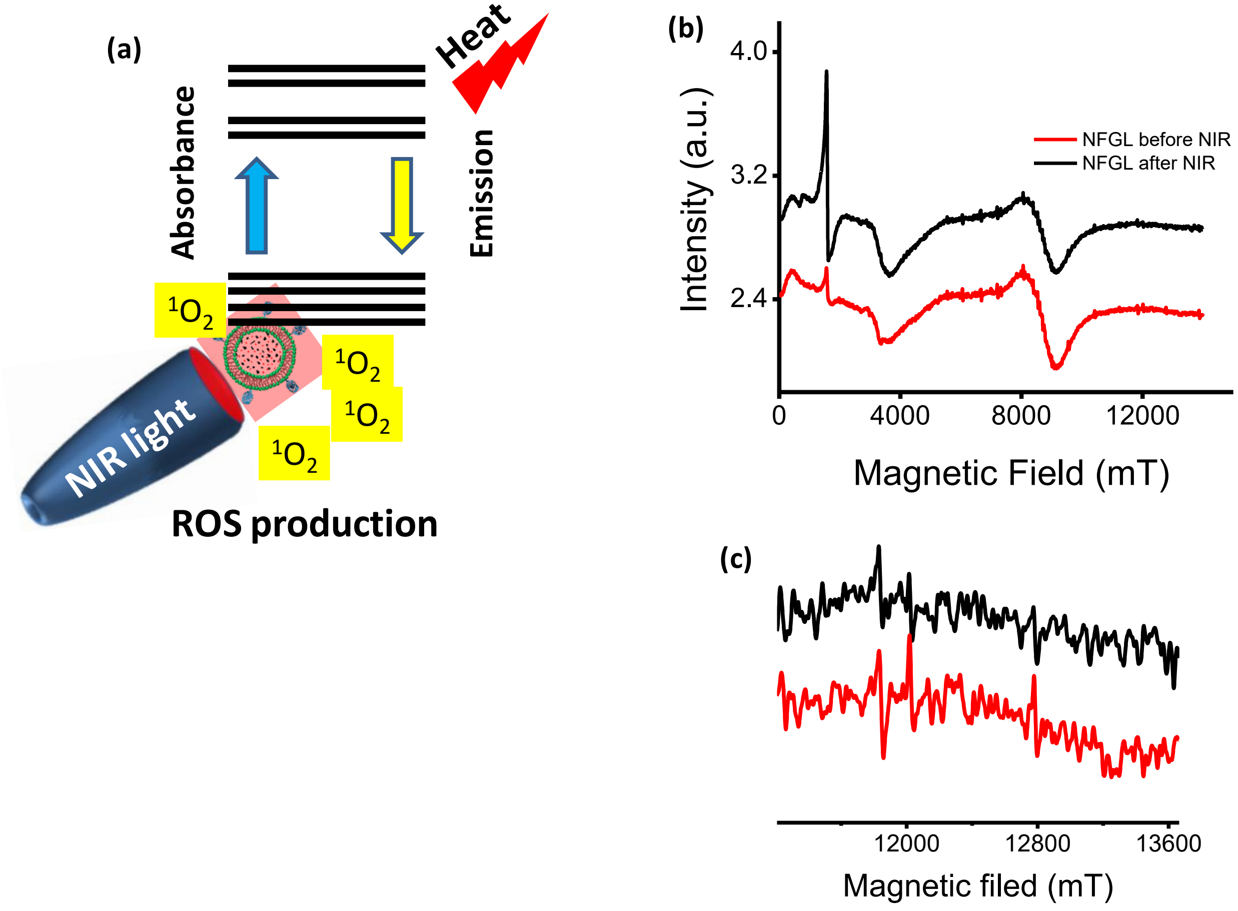

Supplementary Figure 12. (a) Production of reactive oxygen species (ROS) during NIR light exposure, (b, c) electron resonance spectra (ESR) of NFGL nanohybrid before and after NIR light treatment.

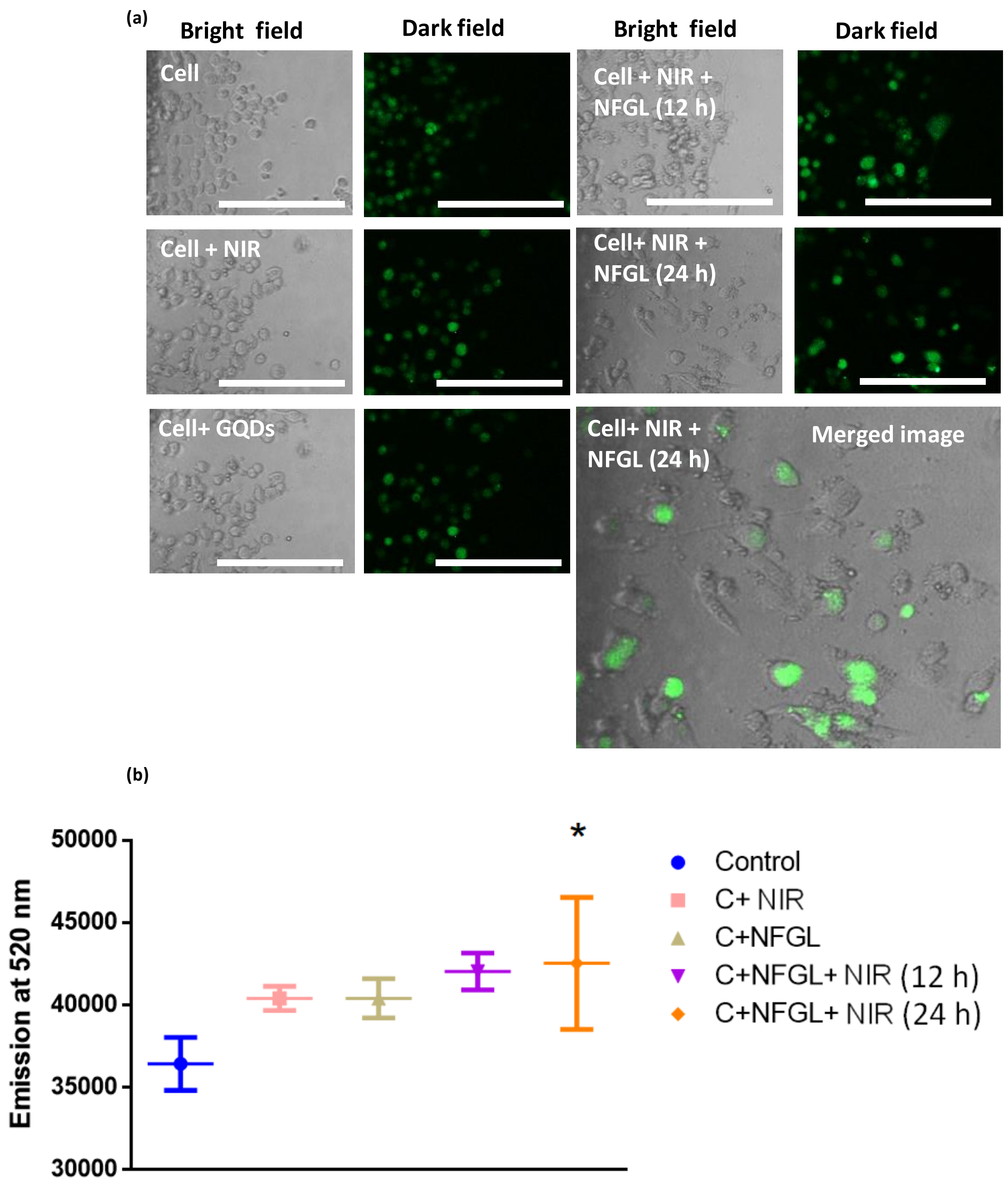

Supplementary Figure 13. (a) Qualitative and (b) quantitative analysis (n = 3, * $P \leq 0.05$) of ROS from 4T1 cancer cells treated NFGL nanohybrid in various conditions. C+NIR is NIR treated cells, C+NFGL is NFGL nanohybrids treated cells, C+NFGL+NIR is NFGL nanohybrids treated cells under NIR light exposure.

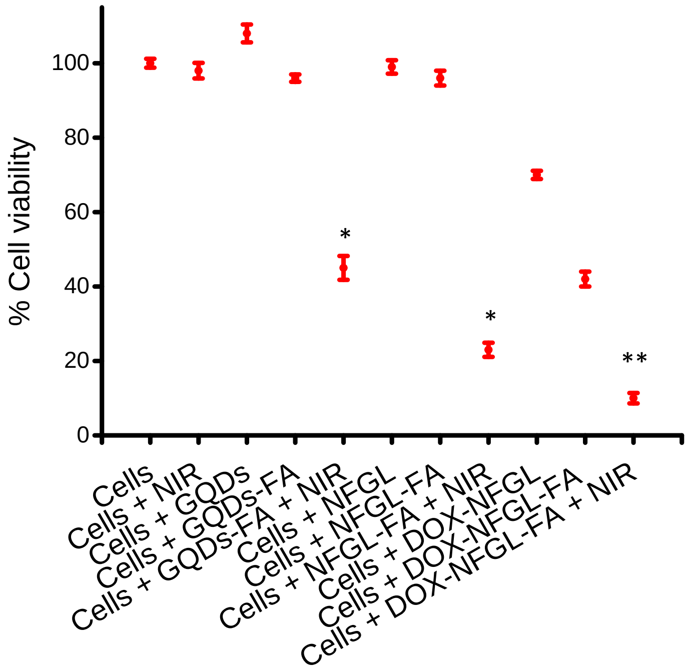

Supplementary Figure 14. Therapeutics measurement of designed NFGL nanohybrids and various components on 4T1 cancer cells in various conditions (% cell viability measured through MTT assay, n = 3, *p< 0.05, **p < 0.01).

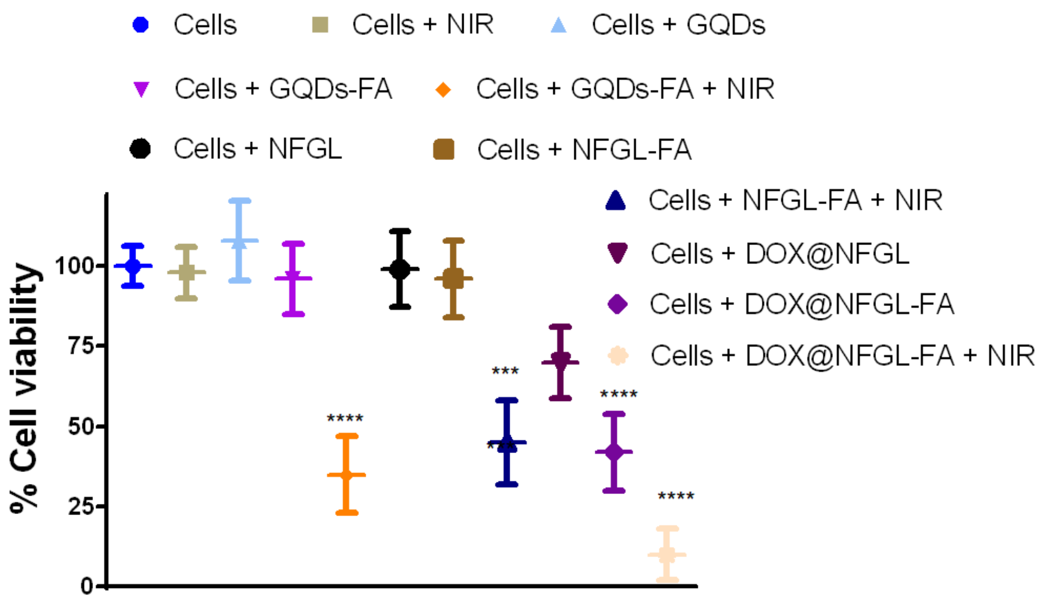

Supplementary Figure 15. Therapeutics measurement of designed NFGL nanohybrids and its various components on MCF-7 cancer cells in various conditions. (% cell viability measured through MTT assay, n = 3, *** P ≤ 0.001 and **** P ≤ 0.0001).

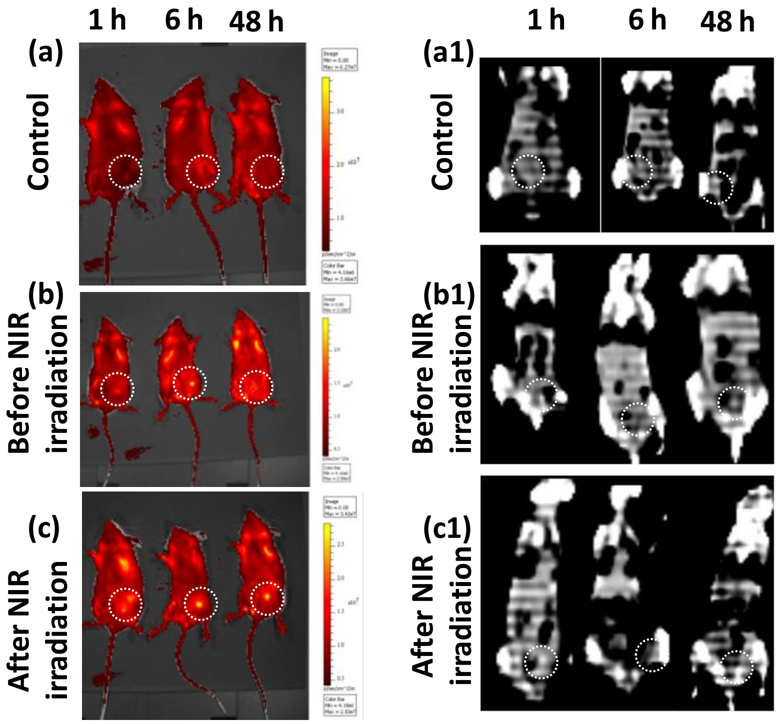

Supplementary Figure 16. Localized tumor diagnosis of 4T1 breast tumor in mice body using NFGL-FA nanohybrid followed by IVIS imaging and X-ray CT imaging modalities before and after NIR light exposure (750 nm, 1 W for 10 minutes) at various time points (1 h, 6 h and 48 h) of post injection. In both imaging modalities, pre-injected mice are considered as control groups.

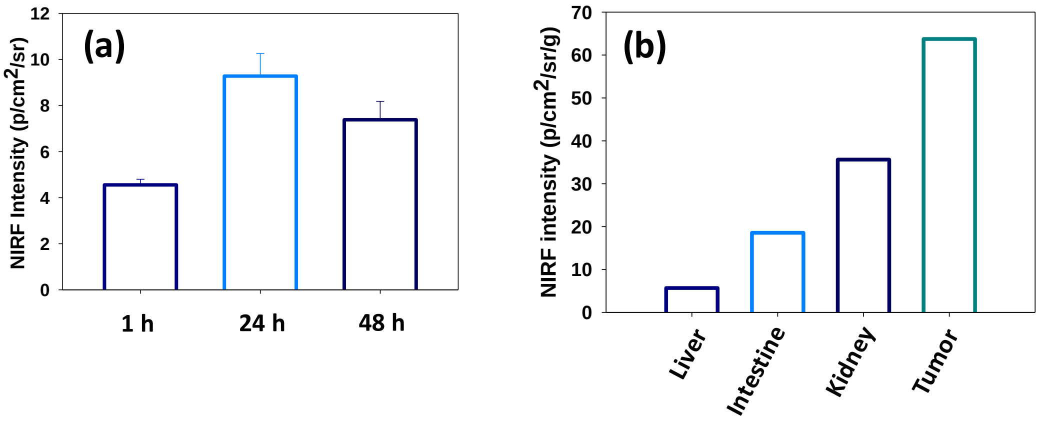

Supplementary Figure 17. (a) Time dependent emission intensity measurement from 4T1 tumor after intravenous injection of NFGL-FA (n = 3). (b) Biodistribution analysis of NFGL-FA after intravenous injection in 4T1 tumor bearing mice.

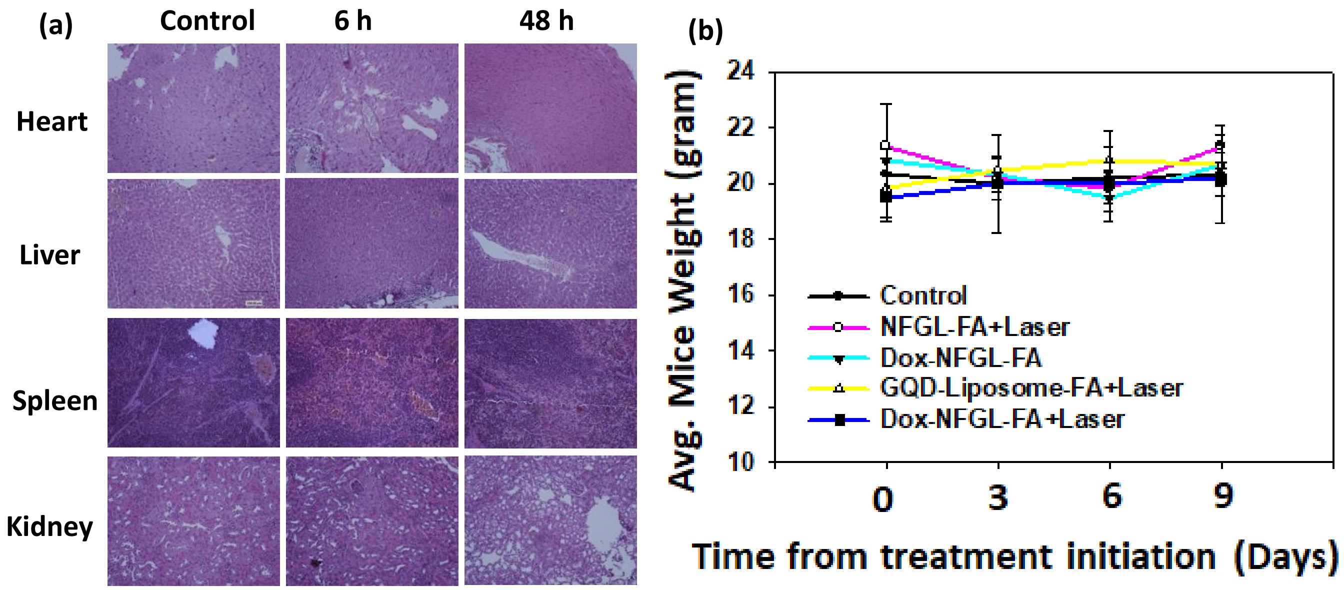

Supplementary Figure 18. (a) Hematoxylin and Eosin (H&E) measurements of major organs at various time of post-injection of NFGL-FA nanohybrids. (b) Body weight measurements of different mice groups during various therapeutic conditions (different formulations of NFGL nanohybrid is injected intravenously in 4T1 tumor bearing mice, n = 3).

	HU values after NIR treatment			HU values before NIR treatment			HU values in Control		
	1 h	6 h	48 h	1 h	6 h	48 h	1 h	6 h	48 h
Tumor	243	278	300	200	233	215			
Heart	140	100	68	110	96	99			
Liver	208	198	123	198	250	245			
Spleen	68	76	118	50	56	63			
Intestine	78	121	154	52	57	120			
Kidneys	42	156	251	39	169	189			

Supplementary Table 1. Quantitative measurements of radiodensity (HU values) in major organs and tumor form X-ray CT imaging with and without NIR exposure experiments and compared with pre-injected mice.

	IVIS intensity (1×10^7) after NIR treatment			IVIS intensity (1×10^7) before NIR treatment			IVIS intensity (1×10^7) in Control		
	1 h	6 h	48 h	1 h	6 h	48 h	1 h	6 h	48 h
Tumor	1.98	2	2.3	1.4	0.88	1.52			
Heart	1.2	0.5	0.01	1	0.80	0.68			
Liver	1.98	0.68	1.3	1	0.85	0.98			
Spleen	0.28	0.81	1.89	0.64	0.89	0.80			
Intestine	0.64	1.18	2.1	0.61	0.38	0.72			
Kidneys	0.18	1.88	1.89	1.22	0.96	1.34			

Supplementary Table 2. Quantitative measurements of IVIS intensity in major organs and tumor with and without NIR exposure experiments and compared with pre-injected mice.

$$\%\ \text{Encapsulation efficiency} = \frac{\text{Total mass of nanohybrid}}{\text{Initial mass of nanohybrid}} \times 100 \qquad \text{Equation 1}$$

Supplementary Equation 1. % Encapsulation efficiency of nanohybrids.
