# Supplementary material for: Liposomal nanotheranostics for multimode targeted in vivo bioimaging and near‐infrared light mediated cancer therapy
Source: Commun Biol. 2020 Jun 5;3:284. doi: 10.1038/s42003-020-1016-z (PMC7275035; doi:10.1038/s42003-020-1016-z)
Supplement: Supplementary file 3 — Description of Additional Supplementary Files [file 42003_2020_1016_MOESM3_ESM.pdf]

Legend of supplementary data

Fig. 4 (a) Absorption spectra of parent Liposome, prepared Graphene Quantum Dots and Gold nanoparticles and Graphene Quantum Dots loaded liposomal nanohybrids named as NFGL at two different time points *viz.*, 0.5 h and 24 h. (b) Photoluminescence spectra of prepared Graphene Quantum Dots, Graphene Quantum Dots encapsulated liposomes and engineered NFGL nanohybrids.

Fig. 5 (a) Contrast measurements of designed Gold nanoparticles and Graphene Quantum Dots loaded liposomal nanohybrids named as NFGL at various concentration (5-100 µg/mL) using a clinical TOSHIBA 64 CT clinical scanner with 5 mm slice thickness and 1 second rotation time compared with parent Liposome. (b) Emission performance of NFGL and compared with liposomes and PBS using *in vivo* imaging system. (c) Time dependent photothermal response of NFGL at 0.5 mg/mL concentration using 750 nm of NIR light irradiation (1 W) compared with parent Liposome (n = 3).

Fig. 6 (b) Observations of produced Reactive Oxygen Species (ROS) during NIR light irradiation when nanohybrids were treated with 4T1 cancer cell lines, ROS are noticed by (2',7'-dichlorofluorescin diacetate, DCFDA) dye staining. (d) % Cell viability of various components of NFGL nanohybrids using 24 h MTT assay at different concentrations (0.1-1 mg/mL, n = 3).

Fig. 8 Time dependent quantitative analysis about the biodistributions of post-injected Gold nanoparticles and Graphene Quantum Dots loaded liposomal nanotheranostics with folic acid functionalization (NFGL-FA) in major organs and tumor measured through (a-c) X-ray CT imaging (n = 3 mice per group) and (d-f) near infrared fluorescence imaging using *in vivo* imaging system (n = 3 mice per group) with and without NIR exposure experiments and compared with pre-injected mice.

Fig. 9 (d, e) Measurements of tumor reduction by tumor volume (mm^3, *$p < 0.05$) and tumor weight (gram, *$p < 0.05$, **$p < 0.01$) analysis (n = 3 mice per group) during various therapeutic conditions using different formulations of NFGL-FA nanotheranostics with and without NIR light exposure (750 nm, 1 W for 10 minutes), and compared with control group of animals (pre-injected and untreated mice).

Fig. 10 (a) % Hemolysis efficacy of liposomes, Gold nanoparticles and Graphene Quantum Dots loaded liposomal nanotheranostics (NFGL) before and after FA attachment at various concentrations (10-200 μg/mL, n = 3). (b) Body weight measurements of post-injected various mice groups (n = 3).

Supplementary Figure 1. Particle size distribution of liposome based nanotheranostics measured through dynamic light scattering (DLS) measurement.

Supplementary Figure 2. Calculated size distribution of graphene quantum dots.

Supplementary Figure 4. Zeta potential measurement of graphene quantum dots (GQDs), polymer stabilized gold nanoparticles (AuNPs), liposomes and NFGL.

Supplementary Figure 5. RAMAN spectra of graphene quantum dots (GQDs) and NFGL nanohybrid.

Supplementary Figure 6. Absorbance of NFGL nanohybrid loaded with anticancer drug doxorubicin hydrochloride in various conditions.

Supplementary Figure 7. Time dependent photothermal transduction measurements of GQDs loaded nanohybrids and NFGL nanohybrids at various concentrations.

Supplementary Figure 8. % Drug release pattern of designed DOX-NFGL nanohybrid.

Supplementary Figure 10. (a, b) FTIR spectra of GQDs-Liposome-FA, NFGL-FA, DOX-NFGL-FA, GQDs-Liposome, NFGL and DOX-NFGL nanohybrids.

Supplementary Figure 12. (b, c) Electron resonance spectra (ESR) of NFGL nanohybrid before and after NIR light treatment.

Supplementary Figure 13. (b) Quantitative analysis (* $P \leq 0.05$) of ROS from 4T1 cancer cells treated NFGL nanohybrid in various conditions. C+NIR is NIR treated cells, C+NFGL is NFGL nanohybrids treated cells, C+NFGL+NIR is NFGL nanohybrids treated cells under NIR light exposure.

Supplementary Figure 14. NIR light mediated in vitro therapeutics efficiencies (% cell viability measured through MTT assay, *p, **p < 0.05, < 0.01) of NFGL nanohybrid and its various components in different conditions.

Supplementary Figure 15. Therapeutics measurement of designed NFGL nanohybrids and various components of NFGL nanohybrid on MCF-7 cancer cells in various conditions. (% cell viability measured through MTT assay, *** $P \leq 0.001$ and **** $P \leq 0.0001$).

Supplementary Figure 17. (a) Time dependent emission intensity measurement from 4T1 tumor after intra-venous injection of NFGL-FA and (b) specific bio-distribution analysis of NFGL-FA after intravenous injection in 4T1 tumor bearing mice.

Supplementary Figure 18. (b) Body weight measurements of different mice groups during various therapeutic conditions (different formulations of NFGL nanohybrid is injected intravenously in 4T1 tumor bearing mice).
