# Supplementary material for: Liposomal nanotheranostics for multimode targeted in vivo bioimaging and near‐infrared light mediated cancer therapy
Source: Commun Biol. 2020 Jun 5;3:284. doi: 10.1038/s42003-020-1016-z (PMC7275035; doi:10.1038/s42003-020-1016-z)
Supplement: Supplementary file 4 — Reporting Summary [file 42003_2020_1016_MOESM4_ESM.pdf]

nature research

Corresponding author(s): Rohit Srivastava (COMMSBIO-19-1711A)

Last updated by author(s): Apr 3, 2020

Reporting Summary

Nature Research wishes to improve the reproducibility of the work that we publish. This form provides structure for consistency and transparency in reporting. For further information on Nature Research policies, see Authors & Referees and the Editorial Policy Checklist.

Statistics

For all statistical analyses, confirm that the following items are present in the figure legend, table legend, main text, or Methods section.

n/a	Confirmed	
☐	☒	The exact sample size (*n*) for each experimental group/condition, given as a discrete number and unit of measurement
☐	☒	A statement on whether measurements were taken from distinct samples or whether the same sample was measured repeatedly
☐	☒	The statistical test(s) used AND whether they are one- or two-sided<br>*Only common tests should be described solely by name; describe more complex techniques in the Methods section.*
☒	☐	A description of all covariates tested
☒	☐	A description of any assumptions or corrections, such as tests of normality and adjustment for multiple comparisons
☐	☒	A full description of the statistical parameters including central tendency (e.g. means) or other basic estimates (e.g. regression coefficient) AND variation (e.g. standard deviation) or associated estimates of uncertainty (e.g. confidence intervals)
☐	☒	For null hypothesis testing, the test statistic (e.g. *F*, *t*, *r*) with confidence intervals, effect sizes, degrees of freedom and *P* value noted<br>*Give P values as exact values whenever suitable.*
☒	☐	For Bayesian analysis, information on the choice of priors and Markov chain Monte Carlo settings
☒	☐	For hierarchical and complex designs, identification of the appropriate level for tests and full reporting of outcomes
☒	☐	Estimates of effect sizes (e.g. Cohen's *d*, Pearson's *r*), indicating how they were calculated

Our web collection on statistics for biologists contains articles on many of the points above.

Software and code

Policy information about availability of computer code

Data collection	No software was used
Data analysis	Statistical analysis was performed using sigma plot 10.0 software and graphs were plotted by using OriginPro 8 software

For manuscripts utilizing custom algorithms or software that are central to the research but not yet described in published literature, software must be made available to editors/reviewers. We strongly encourage code deposition in a community repository (e.g. GitHub). See the Nature Research guidelines for submitting code & software for further information.

Data

Policy information about availability of data

All manuscripts must include a data availability statement. This statement should provide the following information, where applicable:

- Accession codes, unique identifiers, or web links for publicly available datasets
- A list of figures that have associated raw data
- A description of any restrictions on data availability

Supporting data for the present study are available within this article the Supplementary Information file, and all data information are available from the authors on their reasonable request.

Field-specific reporting

Please select the one below that is the best fit for your research. If you are not sure, read the appropriate sections before making your selection.

☒ Life sciences ☐ Behavioural & social sciences ☐ Ecological, evolutionary & environmental sciences

For a reference copy of the document with all sections, see nature.com/documents/nr-reporting-summary-flat.pdf

Life sciences study design

All studies must disclose on these points even when the disclosure is negative.

Sample size	Experimental protocols with samples of animals (n=3 per group, total n=5 treatment groups) on Balb/c mice were approved by Institutional Animal Ethical Committee (IAEC) of National Centre for Cell Science, Pune, India (NCCS, Pune). The IAEC allow us to conduct the animals experiments as per institute guidelines according to National Centre for Cell Science, Pune, India's laws. IAEC's laws approved that all experiments were performed in the completion with the guidelines of IAEC research program under B318 project.
Data exclusions	No data was excluded.
Replication	Experimental protocols includes the n=3 per group, total n=5 treatment groups of Balb/c mice were approved by Institutional Animal Ethical Committee (IAEC) of National Centre for Cell Science, Pune, India (NCCS, Pune). All experiments were repeated three times (n=3) and all attempts were successful.
Randomization	For the tumor imaging and tumor reduction studies, 4T1 tumor-bearing mice were randomized into the five treatment groups (n=3 animals per group).
Blinding	For all the experimental studies (microscopic, spectroscopic, in vitro, drug release kinetics, photo-thermal transduction analysis) we were blinded for all parts of the experiments and various sets.

Reporting for specific materials, systems and methods

We require information from authors about some types of materials, experimental systems and methods used in many studies. Here, indicate whether each material, system or method listed is relevant to your study. If you are not sure if a list item applies to your research, read the appropriate section before selecting a response.

Materials & experimental systems

n/a	Involved in the study	
☒	☐	Antibodies
☐	☒	Eukaryotic cell lines
☒	☐	Palaeontology
☐	☒	Animals and other organisms
☒	☐	Human research participants
☒	☐	Clinical data

Methods

n/a	Involved in the study	
☒	☐	ChIP-seq
☒	☐	Flow cytometry
☒	☐	MRI-based neuroimaging

Eukaryotic cell lines

Policy information about cell lines

Cell line source(s)	All cell lines were from American Type Culture Collection (ATCC) cultured at National Centre for Cell Science, Pune
Authentication	All cell lines were authenticated by National Centre for Cell Science, Pune
Mycoplasma contamination	Testing was done by Tumor Biology, Angiogenesis and Nanomedicine Research Laboratory, National Center for Cell Science, Pune
Commonly misidentified lines (See ICLAC register)	NA

Animals and other organisms

Policy information about studies involving animals; ARRIVE guidelines recommended for reporting animal research

Laboratory animals	6 weeks old female Balb/c mice were used for the present study

Wild animals	NA
Field-collected samples	NA
Ethics oversight	Experimental protocols on Balb/c mice were approved by Institutional Animal Ethical Committee (IAEC) of National Centre for Cell Science, Pune, India (NCCS, Pune). The IAEC allow us to conduct the in vivo experiments as per institute guidelines according to National Centre for Cell Science, Pune, India's laws. IAEC's laws approved that all experiments were performed in the completion with the guidelines of IAEC research program under B318 project.

Note that full information on the approval of the study protocol must also be provided in the manuscript.
